# Cone beam computed tomography for the assessment of linear scleroderma of the face

**DOI:** 10.1186/s12969-017-0218-5

**Published:** 2018-01-03

**Authors:** C. Di Giovanni, S. Puggina, A. Meneghel, F. Vittadello, G. Martini, F. Zulian

**Affiliations:** 1 Department of Woman and Child Health, University of Padua, Via Giustiniani 3, 35128 Padova, Italy; 2 Affidea Group, Unix Radiology Service, Piove di Sacco, Italy

**Keywords:** Localized scleroderma, Morphea, Scleroderma en coup de sabre, Linear scleroderma of the face, Cone beam computed tomography, Outcome measure

## Abstract

**Background:**

To date, standardized methods for assessing the disease progression of linear scleroderma of the face (LSF) are lacking.

**Objectives:**

We investigated whether Cone Beam Computed Tomography (CBCT) may represent a reliable tool for assessing linear scleroderma of the face (LSF).

**Methods:**

Ten patients with LSF and five age-matched controls underwent CBCT assessment. The transverse sections at three anatomic levels of the maxillofacial bones were analyzed. Measurements of soft tissue and total thickness of both affected and unaffected side of the face were made by a standardized methodology. Six raters evaluated CBCTs twice and blindly one from the other. The intra- and inter-rater reliability was assessed by the Intraclass Correlation Coefficient (ICC).

**Results:**

CBCT was fast and well tolerated by the patients. The inter-rater concordance for the total thickness was excellent, mean ICC 0.75 for patients, 0.89 for controls. The mean ICC for soft tissue thickness was 0.49 for patients, 0.66 for controls. 58.3% of the measurements for patients and 91.2% of those for controls showed excellent ICC results (≥ 0.75). The intra-rater concordance resulted optimal (ICC 0.77–0.99).

**Conclusions:**

CBCT is a reliable technique to assess skin and bony changes of LSF.

Juvenile localized scleroderma (JLS) is often difficult to assess, both at disease onset and during the disease course [1–3]. When the disease involves the face, as in linear scleroderma of the face (LSF) subtype, clinical scores, such as the LoScat [4, 5] and other tools such as infrared thermography [6], laser doppler flowmetry [7], doppler ultrasound [8, 9], computerized skin scoring [10] and MRI [11] have significant limitations in monitoring the disease course.

The Cone Beam Computed Tomography (CBCT) scanner uses a 2D detector and a cone-shaped x-ray beam which allows a scan of the region of interest reproducing a digital volume, thus providing both 2D and 3D images (12). CBCT is less costly and the radiation dose is considerably less than a traditional computed tomography (CT) and has a better spatial resolution [12]. For this reason it has a wide range of applications in pediatric dentistry and maxillofacial surgery and has also been applied for the evaluation of the odontostomatologic involvement in LSF [13].

We investigated whether CBCT may represent a potential tool for the assessment of LSF.

## Patients and methods

Patients with the diagnosis of LSF [1], and followed at our Pediatric Rheumatology Centre entered the study. The control group consisted of healthy age-matched subjects with no evident facial asymmetry who needed CBCT for dental or orthodontic purposes.

Maxillofacial CBCT was performed with a New Tom equipment according with the following parameters: number of slices: 606, axial thickness: 0.250 mm, air kerma 2.77 mGy, DAP 507.56 mGy/cm^2^, CTDIw 2.41 mGy, CTDIvol 2.41 mGy.

Information about the two half-face images were obtained in order to measure the total thickness (TT) and the soft tissue thickness (STT) of standardized segments and planes of the maxillofacial bones.

Before starting the measurements, each CBCT image was oriented in multi planar reconstruction (MPR) mode with the aim of refining the precision of the measurements. We ensured that the frontal plane was perpendicular to the temporal bones and the sagittal plane was perpendicular to the hard palate.

**Fig. 1:**
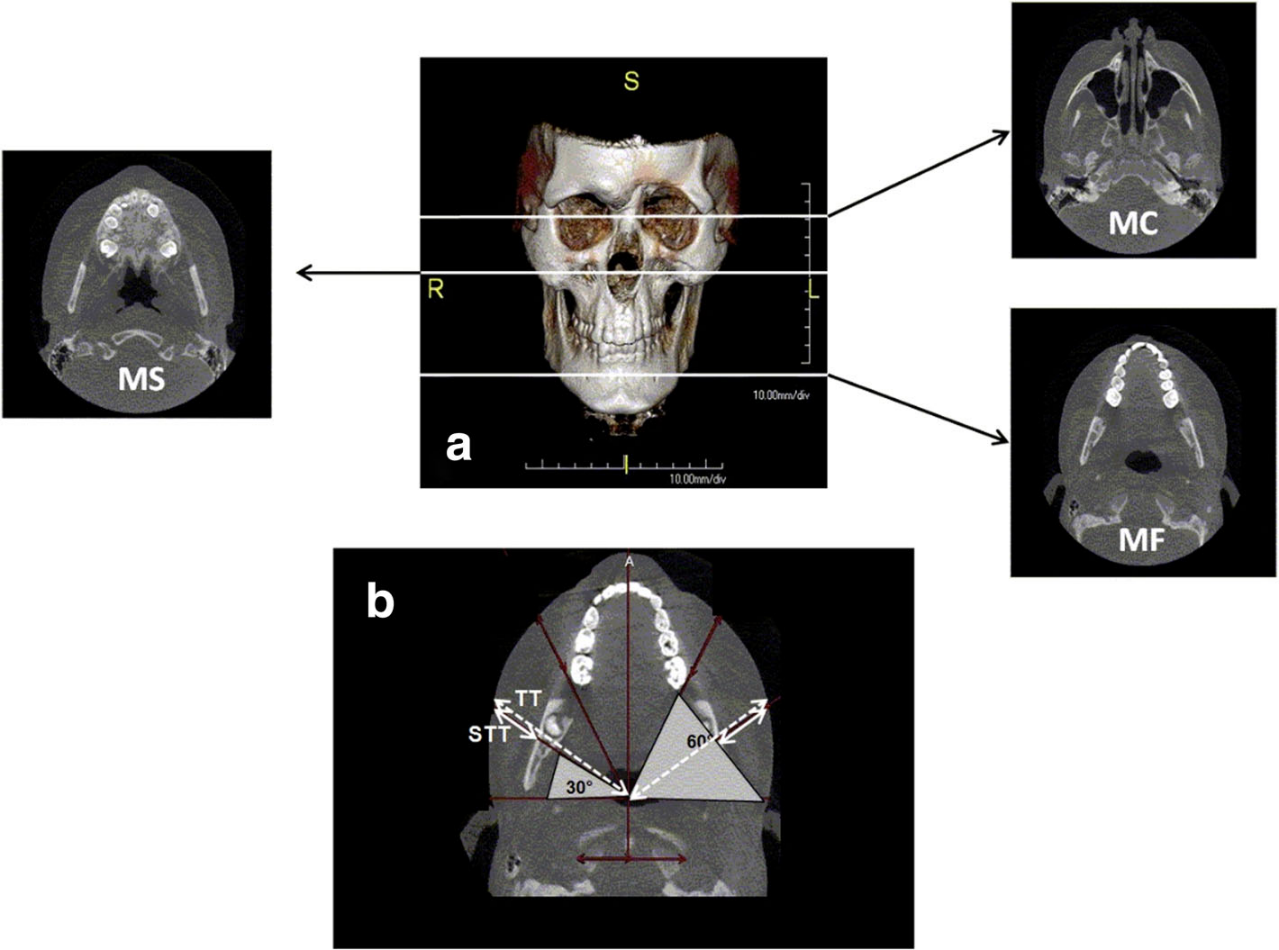
**a** 3D–Cone Beam Computed Tomography (CBCT) showing the levels of the three transverse sections (mandibular condyle, MC, maxillary sinus MS, mandibular foramen MF) selected for the comparison of affected and unaffected side. **b** Example of measurement of total thickness (TT) and soft tissue thickness (STT) at 30° and 60° of the MF section in both affected and unaffected side of the face

In order to include the entire face volume, three main CBCT transverse sections were identified and named by anatomical references as the upper margin of the mandibular condyle (MC), the floor of the maxillary sinus (MS) and the mandibular foramen (MF) (Fig. 1).

Measurements of both affected and unaffected side of the face were made on standardized CBCT sections according to defined anatomical references. At first, a front-to-back and lateral-lateral axis of symmetry were identified. From the intersection axes, an origin point was generated. From this point, 30° and 60° lines, crossing bony and soft structures, were drawn. For each given degree, the soft tissue thickness (STT) and the total thickness (TT), including both bony and soft tissue, of the right and left side were calculated by using the software Onis 2.4 free edition and expressed in millimeters and decimals (Fig. 1). Twenty-four measures for each subject were therefore obtained.

Six raters, all physicians, evaluated 10 LSF patients’ and 5 controls’ CBCTs blindly one from the other. Three raters were experienced with the use of the measurement method while the three not experienced received a two hour training session before starting the measurements. Patients’ CBCTs have been analyzed twice after a time interval of one week in order to evaluate the intra-rater variability which was assessed by the repeatability coefficient with 95% limits of confidence [14].

To assess the inter-rater reliability, we used the intra-class correlation coefficient (ICC) two-way random effect model [15]. The ICC was analysed by calculating and pooling the standard deviations (SD) of the measurements recorded on each subject. The mean SD within patient and variance of all measurements on a single patient were calculated, providing a quantitative assessment of inter-observer reliability. As relative measure of dispersion, the coefficient of variation was calculated for each patient and for each factor. ICC values ranging 0.75–1 were considered signs of excellent reliability, ICC ranging 0.4–0.74 signs of good reliability [16]. The 95% confidence interval (CI) defined the degree of significance and the accuracy of the estimate. All analyses were performed by using IBM SPSS (Version 18.0).

## Results

Ten patients with LSF (7 Parry Romberg Syndrome-PRS, 3 En Coupe De Sabre-PRS), aged 3–21 years, mean disease duration 1.8 years (range 0.5–8 years) and five age-matched healthy controls underwent CBCT assessment. As each patient was independently evaluated twice by each examiner, a set of 48 measures (24 in the first round and 24 in the second one) were obtained. Each examiner also performed 240 measures in 5 controls.

The inter-rater concordance for the total thickness was excellent with mean ICC 0.75 (SD 0.16) for patients (Table 1) and 0.89 (SD 0.09) for controls. The mean ICC for the soft tissue thickness was 0.49 (SD 0.24) for patients and 0.66 (SD 0.28) for controls, respectively. 58.3% of the measurements for patients and 91.2% of those for controls showed excellent ICC results.

**Table 1:** Inter-rater variability of CBCT scan measures

		Soft Tissue Thickness			Total Thickness		
Anatomic Section	Radial Measure	ICC	95% CI	p	ICC	95% CI	p
Mandibular condyle (MC)	Right 30°	0.413	0.15–0.75	.000	0.902	0.78–0.97	.000
	Right 60°	0.543	0.26–0.82	.000	0.937	0.85–0.98	.000
	Left 30°	0.264	0.27–0.64	.013	0.833	0.66–0.95	.000
	Left 60°	0.484	0.21–0.64	.000	0.891	0.76–0.97	.000
Maxillary sinus (MS)	Right 30°	0.693	0.44–0.89	.000	0.458	0.19–0.77	.000
	Right 60°	0.095	−0.88 - 0.47	.183	0.698	0.45–0.90	.000
	Left 30°	0.584	0.30–0.86	.000	0.447	0.16–0.79	.000
	Left 60°	0.120	−0.75 - 0.50	.138	0.752	0.52–0.92	.000
Mandibular foramen (MF)	Right 30°	0.676	0.41–0.89	.000	0.883	0.73–0.97	.000
	Right 60°	0.750	0.53–0.92	.000	0.729	0.50–0.91	.000
	Left 30°	0.478	0.19–0.79	.000	0.766	0.55–0.92	.000
	Left 60°	0.810	0.62–0.94	.000	0.756	0.53–0.92	.000
Mean ICC (SD)		0.493 (0.24)			0.754 (0.16)		
ICC > 0.75 no. (%)		2 (16.6)			7 (58.3)		
ICC 0.40–0.74 no. (%)		7 (58.4)			5 (41.7)		
ICC < 0.40 no. (%)		3 (25.0)			0 (0.0)		

As for the soft tissue thickness, the mean ICC was 0.49 (SD 0.24) with 75% of the measurements being good or excellent (Table 1). As shown, the best performance was obtained at the level of the MF and MC sections. The controls showed an even better concordance for the soft tissue measurements with 41.7% with ICC > 0.75 and 41.7% with ICC 0.40–0.74.

The group of three non-experienced physicians reached significant results in 83.3% of the cases, with ICC ranging 0.62–0.98. The group of experts reached significant results in 73% of cases, with an ICC ranging 0.47–0.94.

The intra-rater repeatability coefficient for the total thickness ranged between 0.857 and 0.996, for the soft tissue thickness ranged between 0.864 and 0.973 (Table 2). All the measurements reached significance and no differences were reported between non-experienced and experienced examiners, as well.

**Table 2:** Intra-rater repeatability and limits of agreement of CBCT scan measures

		Soft Tissue Thickness			Total Thickness		
Anatomic Section	Radial Measure	ICC	95% CI	p	ICC	95% CI	p
Mandibular Condyle (MC)	Right 30°	0.994	(0.984–0.997)	0.000	0.996	(0.989–0.998)	0.000
	Right 60°	0.921	(0.814–0.967)	0.000	0.989	(0.972–0.995)	0.000
	Left 30°	0.984	(0.959–0.993)	0.000	0.992	(0.981–0.996)	0.000
	Left 60°	0.903	(0.774–0.96)	0.000	0.978	(0.945–0.991)	0.000
Maxillary Sinus (MS)	Right 30°	0.969	(0.925–0.987)	0.000	0.981	(0.953–0.992)	0.000
	Right 60°	0.968	(0.922–0.987)	0.000	0.965	(0.916–0.986)	0.000
	Left 30°	0.864	(0.694–0.943)	0.000	0.989	(0.972–0.995)	0.000
	Left 60°	0.973	(0.935–0.989)	0.000	0.962	(0.909–0.984)	0.000
Mandibular Foramen (MF)	Right 30°	0.940	(0.856–0.975)	0.000	0.993	(0.982–0.997)	0.000
	Right 60°	0.967	(0.919–0.986)	0.000	0.990	(0.974–0.995)	0.000
	Left 30°	0.939	(0.855–0.975)	0.000	0.857	(0.679–0.94)	0.000
	Left 60°	0.921	(0.813–0.967)	0.000	0.968	(0.922–0.987)	0.000

Maxillofacial CBCT was fast (mean duration 3.6 s) and well tolerated by the patients, even the youngest. The average time needed to conduct the measurements of CBCTs’ imaging ranged from 15 to 25 min.

## Discussion

The assessment of LSF is still a challenging issue as the tools usually applied for the other subtypes of localized scleroderma have shown significant limitations when applied to LSF [3]. Indeed, there are no validated methods to establish when LSF is in full remission in order to get the surgical reconstruction started.

To date, the most used clinical score, the localized scleroderma assessment tool (LoSCAT) [4, 5], aimed to quantify the skin involvement by clinical signs of activity and damage, is not able to detect changes in the deeper layers of the skin nor bone deformities.

More sensitive assessment tools have both advantages and limitations [3]. Infrared thermography and laser doppler flowmetry present with high sensitivity but low specificity in detecting active LS lesions as an increased number of false positive results are reported. Indeed, it has been demonstrated that they are not appropriate to evaluate deep lesions of the face [6, 7]. Doppler ultrasound has also limitations when applied to LSF due to the lack of standardization and the operator/equipment dependence [8, 9]. Magnetic Resonance Imaging (MRI), currently used to detect musculoskeletal involvement and central nervous system abnormalities in LSF [10, 11], is a long-lasting procedure therefore needs sedation for patients aged less than seven years. As evident, none of these tools are either feasible or sensitive enough to detect disease changes of LSF over time.

For these reasons, given the young age of the majority of the patients with LSF and the need of a fast technique for a concomitant bony and soft tissue evaluation, we investigated the potential role of CBCT for the assessment of LSF, in view of a possible future application of this technique for monitoring the disease over time.

CBCT is currently used in dentistry and maxillofacial surgery, with good sensitivity for both soft and bony tissues [12, 14]. It is fast to perform and minimally invasive since it does not require sedation and results in a radiation exposure 20–50 times lower than a conventional CT [17, 18].

In the present study, we found very good inter-rater concordance for the total thickness, among the assessors, with mean ICC value of 0.75 for patients (Table 1) and 0.89 for controls. Almost 60% of the measurements for patients and more than 90% for controls showed excellent ICC results. The mean ICC for the soft tissue thickness at the MS level was a bit lower than at MF and MC levels, probably because of the objective difficulty in measuring the distance between the superficial skin and the bone surface at that level. However, the intra-rater ICC at the same level was excellent (Table 2), confirming the validity of this technique, particularly when performed by the same operator.

CBCT scan evaluation was feasible also for non-experienced physicians as measurements were not divergent from the experienced ones, confirming the face validity of the method and the efficacy of preliminary training sessions.

CBCT was fast and well tolerated even by the youngest patients. Once obtained the digital images, the average time needed to conduct the standardized analysis of CBCTs’ imaging ranged from 15 to 25 min.

This is the first application of CBCT for the standardized assessment of LSF. Formerly, we applied this technique for the detection of odontostomatologic abnormalities in patients with LSF [13].

As for the possible limitations of CBCT, besides the radiation exposure, this technique does not allow a reliable evaluation of the forehead region, limiting its application for patients with isolated frontal lesions. In fact, due to its conical X-ray emission, the definition and reproducibility of the scan images result to be low for the forehead area, making it inaccurate for comparing measures overtime. We should underline, however, that in a large international study involving 750 patients with juvenile localized scleroderma, 23% presented with LSF but only very few of them had isolated forehead involvement with no maxillary involvement [2]. Therefore, we can evaluate with CBCT the vast majority of patients with LSF. For the few with isolated forehead lesions, as in the initial phase of ECDS, sequential clinical photography and computerized skin scoring can be used with quite good results [10].

In conclusion, we showed that CBCT represents a reliable and relatively safe technique to assess and quantify the disease involvement in LSF. It is fast to be performed and covers an important clinical gap in the clinical practice, not only for the disease assessment but also for issues related to facial reconstructive procedures. In fact, to date, no technique has been able to define the remission status of LSF and the timing for reconstructive plastic surgery procedures. A prospective study on sensitivity to change and disease progression definition by using sequential CBCT checks is ongoing and the preliminary results obtained so far are promising.

## Abbreviations

CBCT: Cone Beam Computed Tomography
ICC: Intraclass Correlation Coefficient
LoSCAT: Localized scleroderma assessment tool
LSF: Linear scleroderma of the face
MC: Mandibular condyle
MF: Mandibular foramen
MPR: Multi planar reconstruction
MRI: Magnetic Resonance Imaging
MS: Maxillary sinus
SD: Standard deviations
STT: Soft tissue thickness
TT: Total thickness
